# Common diagnostic biomarkers and molecular mechanisms of *Helicobacter pylori* infection and inflammatory bowel disease

**DOI:** 10.3389/fimmu.2024.1492810

**Published:** 2024-12-05

**Authors:** Minglin Zhang, Tong Liu, Lijun Luo, Yi Zhang, Qijiao Chen, Fen Wang, Yuxin Xie

**Affiliations:** ^1^ Department of Gastroenterology, The Third Xiangya Hospital, Central South University, Changsha, Hunan, China; ^2^ Department of General Surgery, Zhongshan Hospital of Traditional Chinese Medicine Affiliated to Guangzhou University of Traditional Chinese Medicine, Zhongshan, Guangdong, China; ^3^ School of Medical Laboratory Science, Hebei North University, Zhangjiakou, Hebei, China; ^4^ Department of General Surgery, The First People's Hospital of Qingzhen City, Guiyang, Guizhou, China; ^5^ Department of Infectious Diseases, Affiliated Hospital of Zunyi Medical University, Zunyi, Guizhou, China

**Keywords:** bioinformatics analysis, *Helicobacter pylori*, inflammatory bowel disease, diagnostic biomarkers, molecular mechanism

## Abstract

**Background:**

*Helicobacter pylori* (*H. pylori*) may be present in the intestinal mucosa of patients with inflammatory bowel disease (IBD), which is a chronic inflammation of the gastrointestinal tract. The role of *H. pylori* in the pathogenesis of IBD remains unclear. In this study, bioinformatics techniques were used to investigate the correlation and co-pathogenic pathways between *H. pylori* and IBD.

**Methods:**

The following matrix data were downloaded from the GEO database: *H. pylori*-associated gastritis, GSE233973 and GSE27411; and IBD, GSE3365 and GSE179285. Differential gene analysis was performed via the limma software package in the R environment. A protein−protein interaction (PPI) network of DEGs was constructed via the STRING database. Cytoscape software, through the CytoHubba plugin, filters the PPI subnetwork and identifies Hub genes. Validation of the Hub genes was performed in the validation set. Immune analysis was conducted via the CIBERSORT algorithm. Transcription factor interaction and small molecule drug analyses of the Hub genes were also performed.

**Results:**

Using the GSE233973 and GSE3365 datasets, 151 differentially expressed genes (DEGs) were identified. GO enrichment analysis revealed involvement in leukocyte migration and chemotaxis, response to lipopolysaccharides, response to biostimulatory stimuli, and regulation of interleukin-8 (IL-8) production. Ten Hub genes (TLR4, IL10, CXCL8, IL1B, TLR2, CXCR2, CCL2, IL6, CCR1 and MMP-9) were identified via the PPI network and Cytoscape software. Enrichment analysis of the Hub genes focused on the lipopolysaccharide response, bacterial molecular response, biostimulatory response and leukocyte movement. Validation using the GSE27411 and GSE179285 datasets revealed that MMP-9 was significantly upregulated in both the *H. pylori* and IBD groups. The CIBERSORT algorithm revealed immune infiltration differences between the control and disease groups of IBD patients. Additionally, the CMap database identified the top 11 small molecule compounds across 10 cell types, including TPCA-1, AS-703026 and memantine, etc.

**Conclusion:**

Our study revealed the co-pathogenic mechanism between *H. pylori* and IBD and identified 10 Hub genes related to cellular immune regulation and signal transduction. The expression of MMP-9 is significantly upregulated in both *H. pylori* infection and IBD. This study provides a new perspective for exploring the prevention and treatment of *H. pylori* infection and IBD.

## 1 Introduction

*Helicobacter pylori* (*H. pylori*) is a gram-negative, microaerophilic, spiral-shaped bacterium that infects approximately 50% of the global population and is classified as a Group I human carcinogen. The association between the gastric microbiome and *H. pylori* plays a significant role in the development and progression of gastric cancer (GC) (1, 2). *H. pylori* infection is closely associated with chronic gastritis, peptic ulcers, GC and mucosa-associated lymphoid tissue (MALT) lymphoma. *H. pylori* infection can cure chronic active gastritis and peptic ulcer disease, reduce the risk of peptic ulcer bleeding in aspirin users, and lower the risk of GC in infected individuals (3, 4). Research has shown that *H. pylori* infection increases the risk of developing colorectal cancer (CRC) (5, 6), and reduces the risk of inflammatory bowel disease (IBD) (7, 8). In conclusion, *H. pylori* may be related to IBD, and the specific mechanism needs to be further studied.

IBD is a chronic, recurrent inflammatory disease of the gastrointestinal tract that includes ulcerative colitis (UC) and Crohn’s disease (CD) (9). The main gastrointestinal symptoms of IBD include abdominal pain, diarrhea, bloody stools and weight loss (10). Currently, the aetiology and pathogenesis of IBD are not fully understood. Some studies have shown that genetic and environmental factors, gut microbiota imbalance, intestinal mucosal immune responses, and damage to the intestinal mucosal barrier play important roles in IBD (11–14). Multiple studies have shown that *H. pylori* infection is associated with IBD and that *H. pylori* may influence the immunoregulatory functions related to IBD (8, 15, 16). However, the specific molecular mechanism is still unclear.

In this study, we used bioinformatics methods to comprehensively analyze the relationship between *H. pylori* and IBD on the basis of sequencing data of the two diseases from databases. By analyzing the coexpression of DEGs via GO and KEGG analyses, identifying candidate Hub genes (core genes coexpressed between *H. pylori* and IBD), predicting small-molecule drugs and performing transcription factor analysis, we explored the potential pathogenic mechanisms common to the two diseases.

## 2 Methods

### 2.1 Matrix data download and analysis

In accordance with the previous literature on IBD selection strategies (17–19), through the search of *H. pylori* datasets in the Gene Expression Omnibus (GEO) database, we identified 4 transcriptome datasets, including *H. pylori* (GSE233973 and GSE27411) and IBD (GSE3365 and GSE179285), and collected basic clinical information (sample size, GEO_accession, Type, Sample_organism, Diagnosis, Age, Race, Gender and Country; for further details (**Supplementary Table 1**). First, we analyzed the data from the GSE3365 and GSE233973 datasets and discovered a large number of coexpressed DEGs. We used R language for the generation analysis of the GSE3365 and GSE233973 datasets and verified the Hub genes of the GSE27411 and GSE179285 datasets.

### 2.2 Screening of coexpressed DEGs

For the GSE3365 and GSE233973 datasets, the limma software package was used to identify DEGs in the data in the R environment, and the screening criteria were P.adj. value < 0.05 and |LogFC| > 0.585.

### 2.3 Functional enrichment analysis of DEGs

GO, KEGG and GSEA (20, 21) pathway enrichment analyses were performed via R packages such as org.Hs.eg.db, clusterProfiler, enrichplot and enrichment plot. GSEA is used to identify important functional items between *H. pylori* or IBD and control samples and to annotate gene pathways. p < 0.05 indicated that enrichment was statistically significant.

### 2.4 PPI network construction and Hub gene screening

A total of 151 DEGs were input into the STRING platform to delete isolated genes. Cytoscape software was used to screen the Hub genes, and the MCC algorithm was used to calculate the top 10 genes in the PPI network. Finally, the Hub genes were input into the GeneMANIA network tool to analyze the gene coexpression network (22).

### 2.5 Verification of *H. pylori* and IBD Hub genes

In the validation datasets GSE27411 and GSE179285, the ggpubr package in R software was used to verify the expression levels of Hub genes in *H. pylori* and IBD and to determine their diagnostic ability for *H. pylori* and IBD.

### 2.6 Immune infiltration analysis

With the CIBERSORT calculation method (23), the proportion of immune cells in each sample was calculated, as were the expression levels of genes in the 22 immune cells.

### 2.7 CMap analysis to identify small molecule drugs

Connectivity Map (CMap): A tool or method that uses gene expression signatures to link small molecules, genes and diseases. We input the Hub genes into CMap to identify small molecule compounds with potential therapeutic effects and screen for predicted small molecule drugs. The structure of small molecule drugs was screened by PubChem.

### 2.8 Transcription factor regulatory network and differential analysis

TRRUST v2 (TRRUST version 2) is a manually curated database of human and mouse transcriptional regulatory networks (24). The transcription factors that interact with the Hub genes were analyzed via the TRRUST v2 transcriptional regulatory interaction database. The Hub genes were input into the TRRUST database, a transcription factor regulatory network diagram was constructed, and the transcription factors were output. Differential analysis of the transcription factors was performed via the GSE3365 and GSE233973 datasets. In addition, the common databases used for data processing in this article are detailed in **Supplementary Table 9**.

## 3 Results

### 3.1 Identification of DEGs in *H. pylori* and IBD

**Figure 1:**
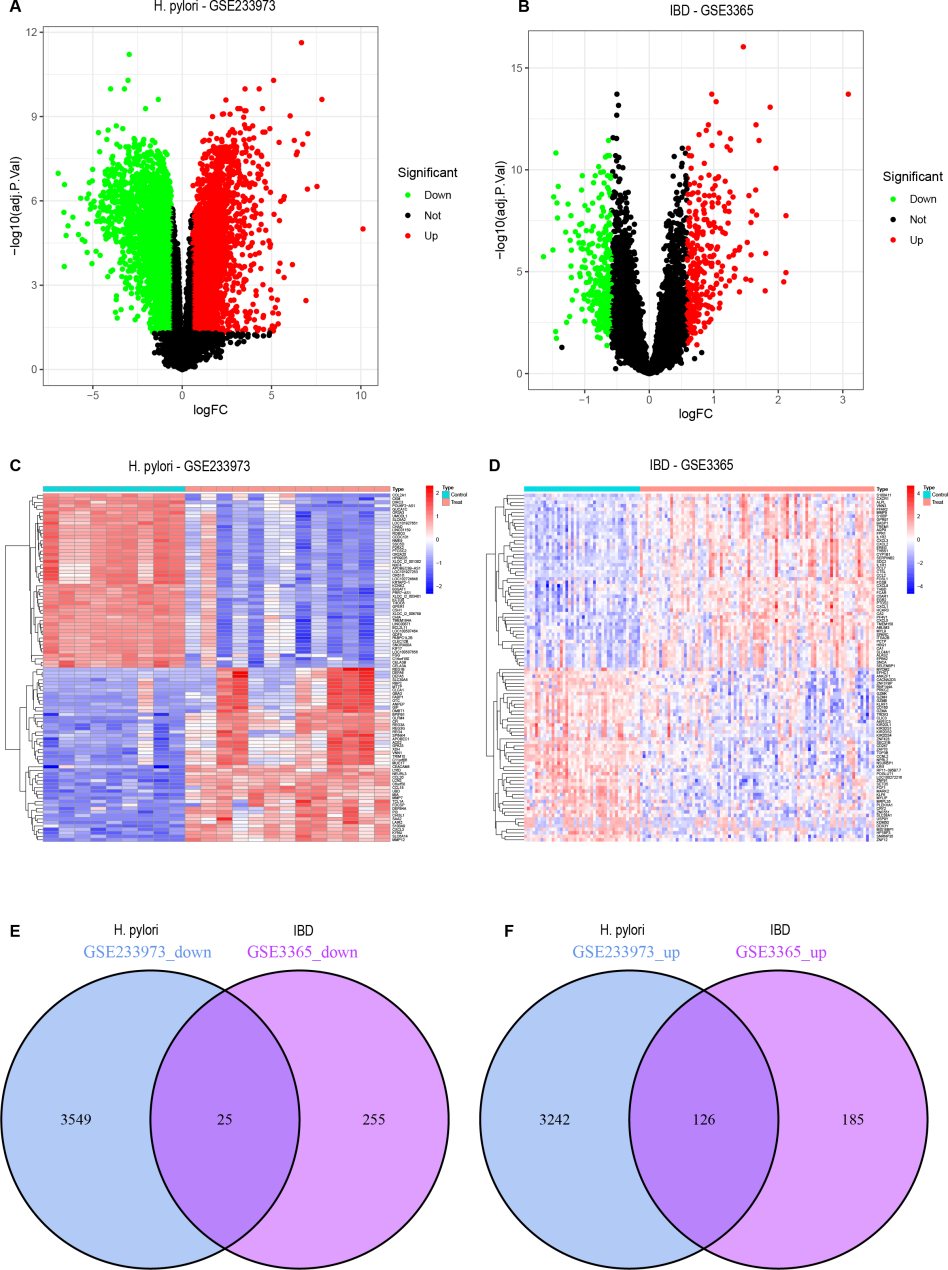
DEGs volcano plots, heatmaps and co-expressed genes. **(A)** Volcano plot representing DEGs from the GSE233973 dataset. **(B)** Volcano plot representing DEGs from the GSE3365 dataset. **(C)** Heatmap representing DEGs from the GSE233973 dataset. **(D)** Heatmap representing DEGs from the GSE3365 dataset. **(E)** Venn diagram of the coexpressed downregulated DEGs shared between the GSE233973 and GSE3365 datasets. **(F)** Venn diagram of the coexpressed upregulated DEGs shared between the GSE233973 and GSE3365 datasets. Red represents upregulated genes, green represents downregulated genes, and grey represents genes with no differential expression.

Through the *H. pylori* dataset (GSE233973) from the GEO database, a total of 6,942 DEGs were identified, as shown in the volcano plot, including 3,368 upregulated genes and 3,754 downregulated genes (**Figure 1A**). The heatmap displays the top 50 most upregulated and downregulated genes (**Figure 1C**), with OLFM4 being the most upregulated gene. From the IBD dataset (GSE3365), a total of 591 DEGs were identified. As shown in the volcano plot, there were 311 upregulated genes and 280 downregulated genes (**Figure 1B**), the heatmap displays 50 differential genes (**Figure 1D**), with SERPINB2 being the most significantly upregulated gene. Finally, 151 overlapping genes were identified between the *H. pylori* and IBD datasets, including 126 upregulated genes and 25 downregulated genes (**Figures 1E, F**). A list of DEGs and common genes can be found in **Supplementary Tables 2**–**4**.

### 3.2 Functional enrichment analysis of DEGs

**Figure 2:**
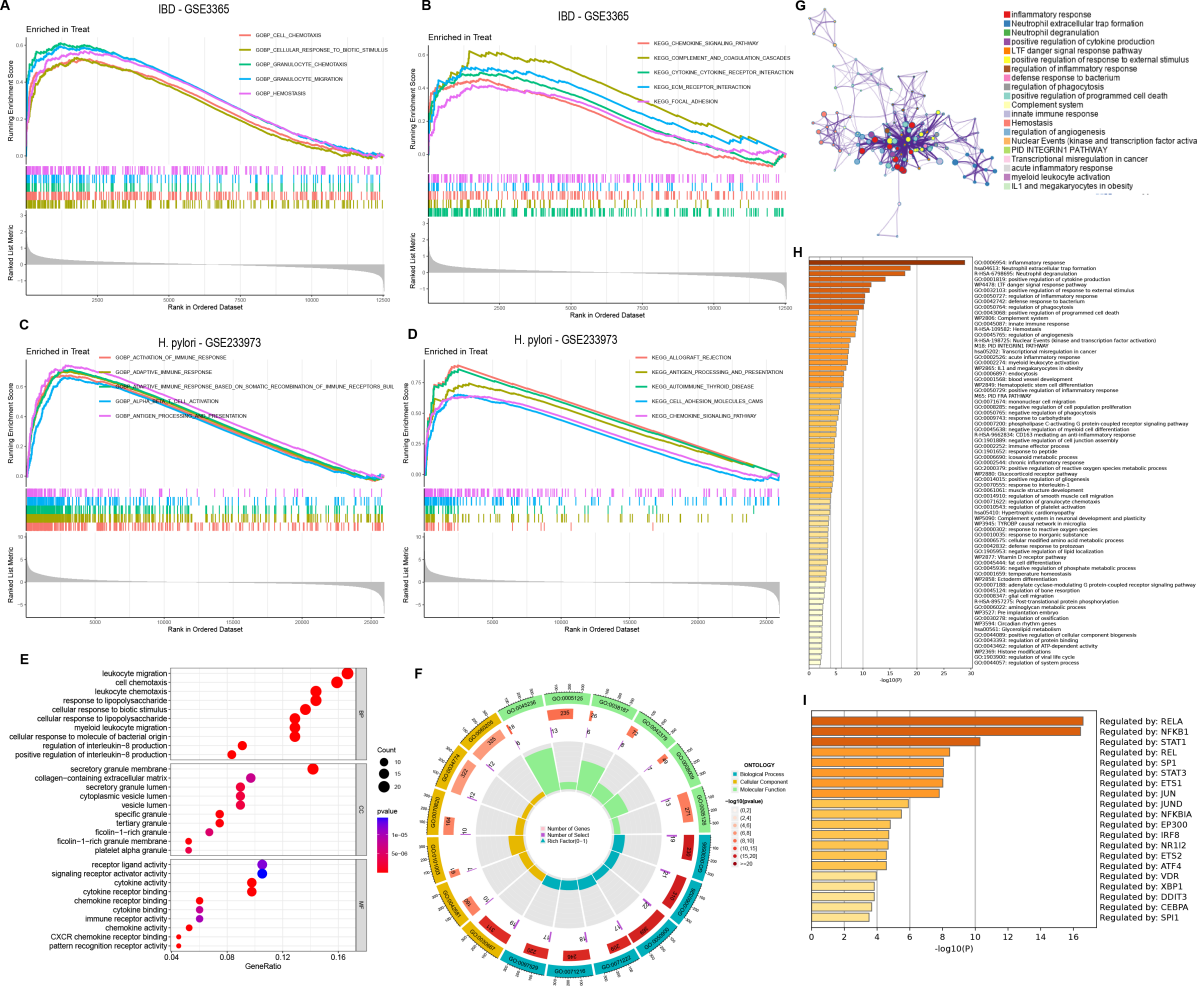
Functional enrichment and pathway enrichment analysis of coexpressed DEGs in *H. pylori* and IBD. **(A–D)** GSEA of coexpressed DEGs, with GO and KEGG enrichment analyses of the GSE3365 and GSE233973 datasets. Treat represents disease group. **(E)** GO analysis of coexpressed DEGs, including BP, biological process; CC, cellular component; MF, molecular function. **(F)** Circular plot of the GO analysis results. **(G)** PPI network analysis of coexpressed DEGs. **(H)** Enrichment analysis of 151 coexpressed DEGs via the Metascape online tool. **(I)** Transcription factor analysis of coexpressed DEGs.

In the functional enrichment analysis, 151 DEGs were coexpressed by *H. pylori* and IBD. By GSEA analysis, in IBD (GSE3365), GO enrichment analysis revealed that these DEGs were enriched mainly in CELL_CHEMOTAXIS, CELLULAR_RESPONSE_TO_BIOTIC_STIMULUS, GRANULOCYTE_CHEMOTAXIS, GRANULOCYTE_MIGRATION and HEMOSTASIS. KEGG enrichment analysis suggested that these DEGs were enriched mainly in CHEMOKINE_SIGNALING_PATHWAY, COMPLEMENT_AND_COAGULATION_CASCADES, CYTOKINE_CYTOKINE_RECEPTOR_INTERACTION, ECM_RECEPTOR_INTERACTION KEGG_FOCAL_ADHESION and other pathways (**Figures 2A, B**). In *H. pylori* (GSE233973), GO enrichment analysis revealed that these DEGs were enriched mainly in ACTIVATION_OF_IMMUNE_RESPONSE, ADAPTIVE_IMMUNE_RESPONSE_BASED_ON_SOMATIC_RECOMBINATION_OF_IMMUNE_RECEPT ORS_BUILT, ALPHA_BETA_T_CELL_ACTIVATION, and ANTIGEN_PROCESSING_AND_PRESENTATION on the equal path. KEGG enrichment analysis indicated that these DEGs were enriched mainly in ALLOGRAFT_REJECTION, ANTIGEN_PROCESSING_AND_PRESENTATION, AUTOIMMUNE_THYROID_DISEASE and CELL_ADHESION_MO LECULES_CAMS, CHEMOKINE_SIGNALING_PATHWAY and other pathways (**Figures 2C, D**). In addition, the enrichment analysis focused on four main aspects: reactions to lipopolysaccharides, leukocyte chemotaxis, cellular chemotaxis and leukocyte migration (**Figures 2E, F**).

Furthermore, to further elucidate the enriched pathways of the DEGs, the MetScape database played a significant role in disease enrichment analysis, interorgan correlation, and transcription factor interactions. We found that different genes in the MetScape database may exhibit different functional group distributions, with the positive regulation of the inflammatory response being the most prominent (**Figure 2G**). Moreover, enrichment analysis based on the MetScape database revealed a common role of inflammation in the pathogenesis of *H. pylori* infection and IBD (**Figure 2H**). Finally, transcription factor analysis of the DEGs revealed RELA, BFKB and STAT1 as the most commonly regulated genes (**Figure 2I**).

### 3.3 Extraction and enrichment analysis of *H. pylori* and IBD Hub genes

**Figure 3:**
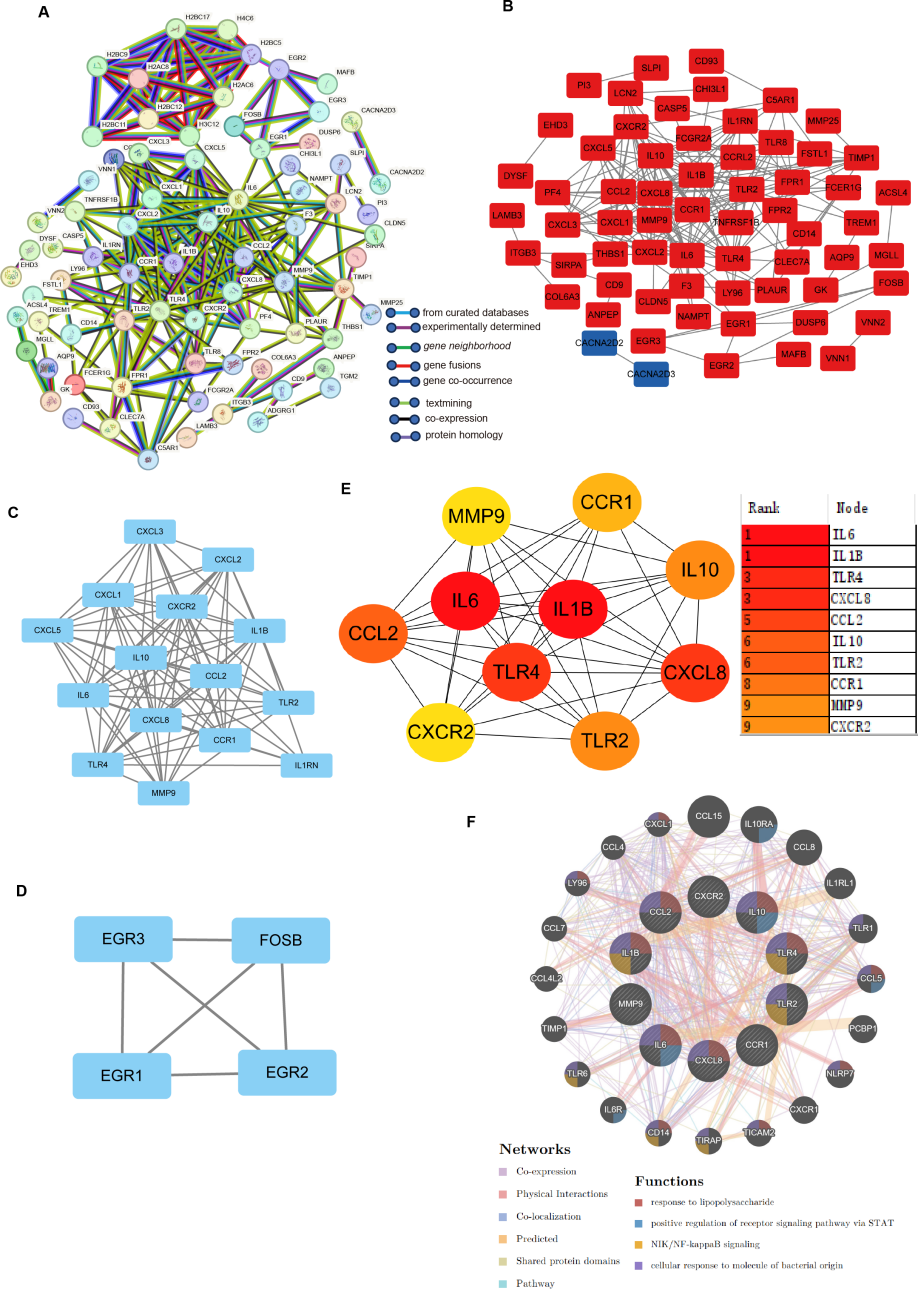
Identification of hub genes and functional interaction network diagram. **(A)** PPI network analysis of DEGs (STRING database). **(B, E)** The top 10 Hub genes identified by the degree and MCC algorithms via the CytoHubba plugin in Cytoscape. **(C, D)** Visualization of the PPI network and important modules and subnetworks. **(F)** GeneMANIA diagram showing the coexpression interactions between the 10 identified shared Hub genes and their neighbouring genes. The colour codes indicate the functions shared by the genes.

PPI analysis of the DEGs coexpressed in *H. pylori* and IBD was performed via the STRING database. To further categorize the DEGs into functional groups, we input the 151 DEGs into the STRING database and removed the independent genes to construct a PPI network diagram (**Figure 3A**). The analysis was conducted via the CytoHubba plugin in Cytoscape software, with the visualization results shown in Cytoscape (**Figure 3B**). On the basis of the visualization results, MCODE analysis yielded two subnetwork diagrams of DEGs, wherein the genes within each subnetwork exhibited strong interconnections (**Figures 3C, D**). The MCC and degree algorithms were used to analyze the PPI network, identifying the top 10 Hub genes: TLR4, IL10, CXCL8, IL1B, TLR2, CXCR2, CCL2, IL6, CCR1 and MMP-9. These 10 Hub genes were identified as candidate diagnostic markers, with IL6 being the most significant within the group (**Figure 3E**; **Supplementary Table 5**). GeneMANIA network analysis revealed the interaction network of the 10 Hub genes, with the outer circle indicating the interaction relationships with the Hub genes (**Figure 3F**).

**Figure 4:**
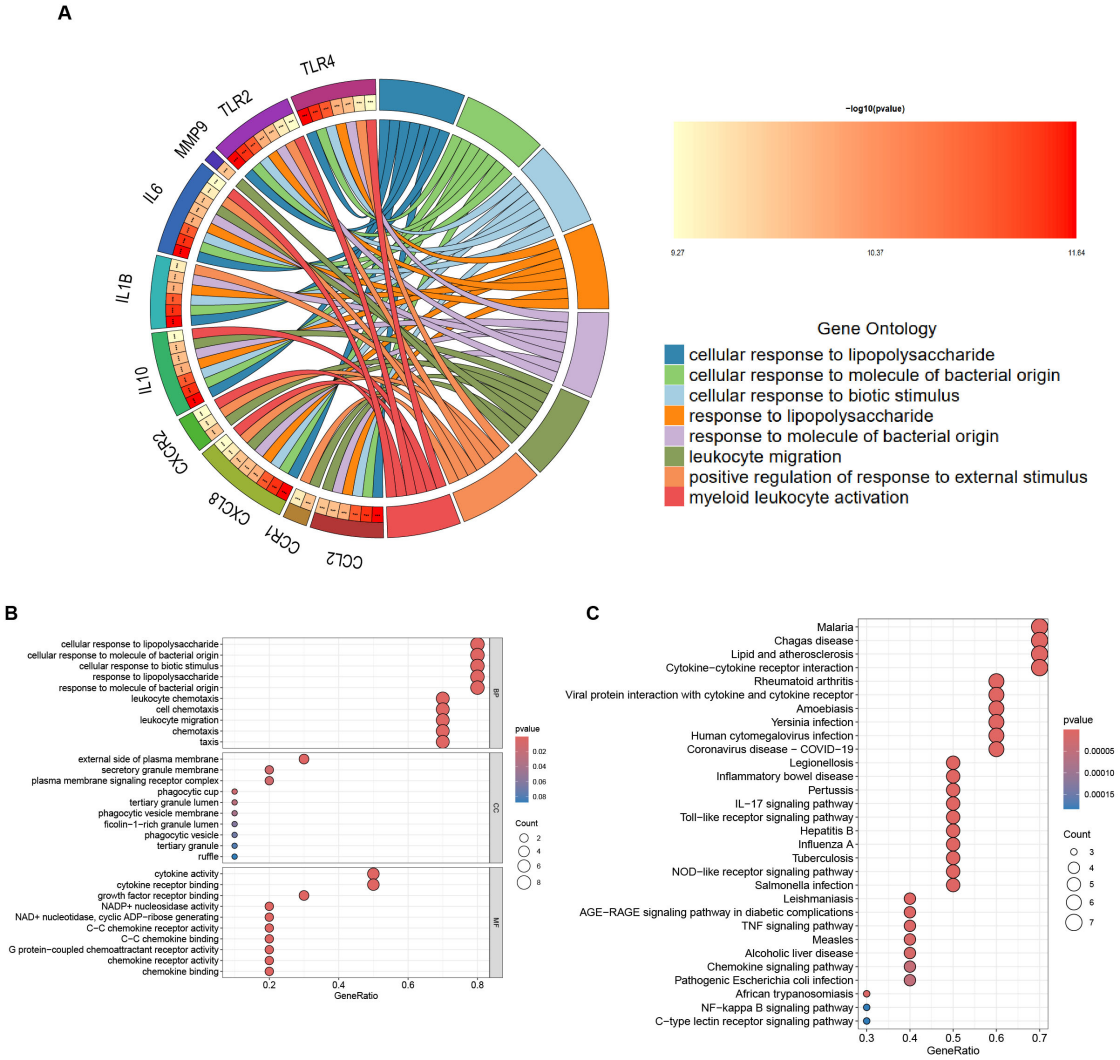
Enrichment Analysis of Hub Genes. **(A)** The circular diagram visualizing the results of the GO enrichment analysis is presented below: the left semicircle denotes Hub genes, which represent gene names, whereas the right semicircle indicates GO terms. The different colours correspond to different GO terms. Notably, the connections between the two semicircles illustrate the enrichment of genes within specific GO terms. ***p < 0.001. **(B)** GO enrichment analysis of the following Hub genes: BP, biological process; CC, cellular component; MF, molecular function. **(C)** KEGG enrichment analysis of the Hub genes.

In the GO functional enrichment analysis, the 10 Hub genes associated with *H. pylori* and IBD were enriched in three key aspects: cellular response to lipopolysaccharide, cellular response to molecule of bacterial origin and cellular response to biotic stimulus (**Figures 4A, B**). Furthermore, KEGG analysis suggested that these Hub genes are mainly enriched in Malaria, Chagas disease, Lipid and atherosclerosis and Cytokine-cytokine receptor interaction, it is particularly noteworthy that the KEGG analysis indicated a significant association between these Hub genes and IBD (**Figure 4C**).

### 3.4 Validation of Hub Genes in *H. pylori* and IBD

**Figure 5:**
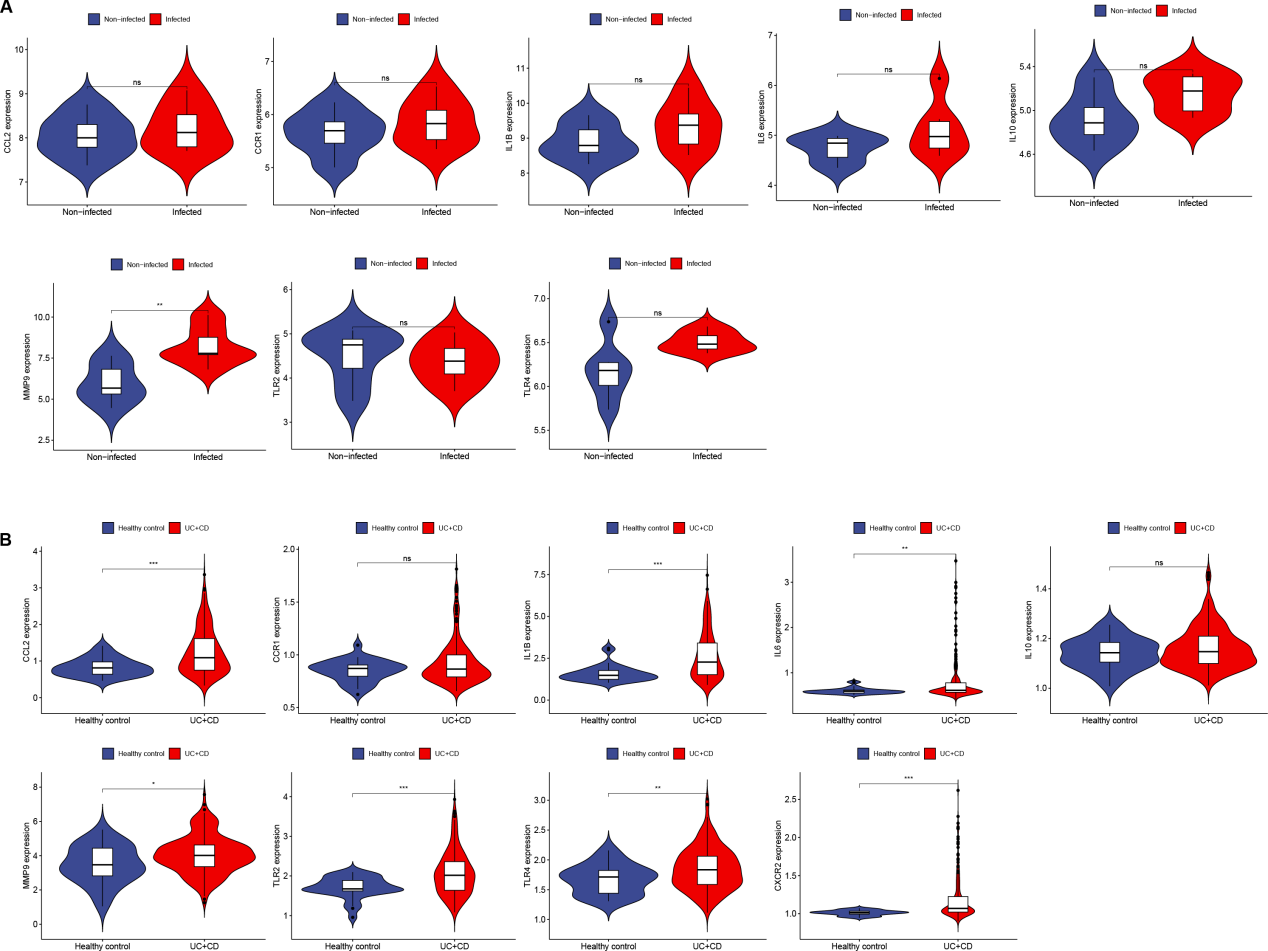
Expression of Hub genes in *H. pylori* and IBD. **(A, B)** Expression of 10 Hub genes in GSE27411 and GSE179285. **(A)** Blue represents noninfected individuals, and red represents *H. pylori* patients. **(B)** Blue represents normal individuals, and red represents UC+CD patients. *p < 0.05; **p < 0.01; ***p < 0.001; ns, no statistical significance.

The Hub genes were validated in the *H. pylori* dataset (GSE27411), revealing upregulated expression of the Hub genes, with MMP-9 showing a significant increase in expression (**Figure 5A**). In the IBD dataset (GSE179285), the Hub genes were also found to be upregulated, with MMP-9, IL6, CCL2, TLR4, TLR2, IL1B and CXCR2 showing significant increases in expression (**Figure 5B**).

### 3.5 Immune infiltration analysis

**Figure 6:**
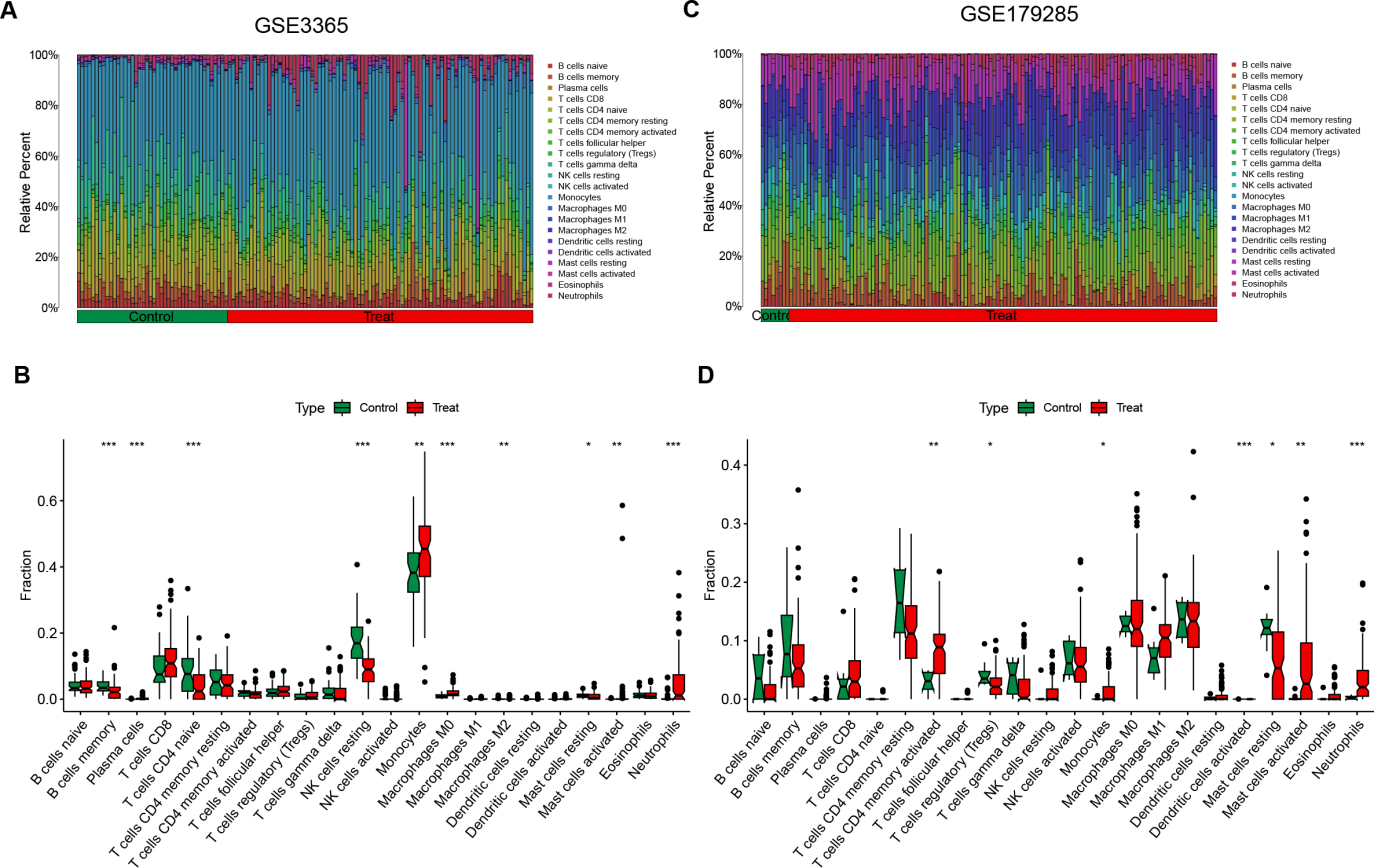
Immune cell infiltration analysis of DEGs in IBD. **(A, C)** These figures represent the extent of infiltration of various immune cells between the IBD disease group and the normal group. **(B, D)** Violin plots depicting the differences in immune-infiltrating cells between the IBD disease group and the normal group. The horizontal axis represents the names of the immune cells, and the vertical axis represents the content of the immune cells. Green represents the normal group, and red represents the disease group. Treat represents IBD disease group. *p < 0.05; **p < 0.01; ***p < 0.001; ns indicates no statistical significance.

Enrichment analysis revealed that inflammatory responses and immune reactions play crucial roles in the development of both diseases. In the IBD datasets (GSE3365 and GSE179285), we investigated different patterns of immune infiltration on the basis of 22 types of immune cells via the CIBERSORT method (**Figures 6A, C**). The distribution of immune cells varied between the two IBD datasets, which may be attributed to differences in sample size and the clinical characteristics of the patients. In IBD, immune cell infiltration was increased in CD8+ T cells, monocytes, M0 macrophages, activated mast cells and neutrophils, whereas memory B cells, resting NK cells and resting mast cells were decreased (**Figures 6B, D**). Owing to the small sample size of the *H. pylori* datasets, no valid results were obtained. Moreover, we reviewed the relevant literature and reported that *H. pylori* is related to the aforementioned immune cells. Therefore, immune responses and inflammatory reactions may occur in both *H. pylori*-infected individuals and individuals with IBD.

### 3.6 Prediction of potential small-molecule drugs

**Figure 7:**
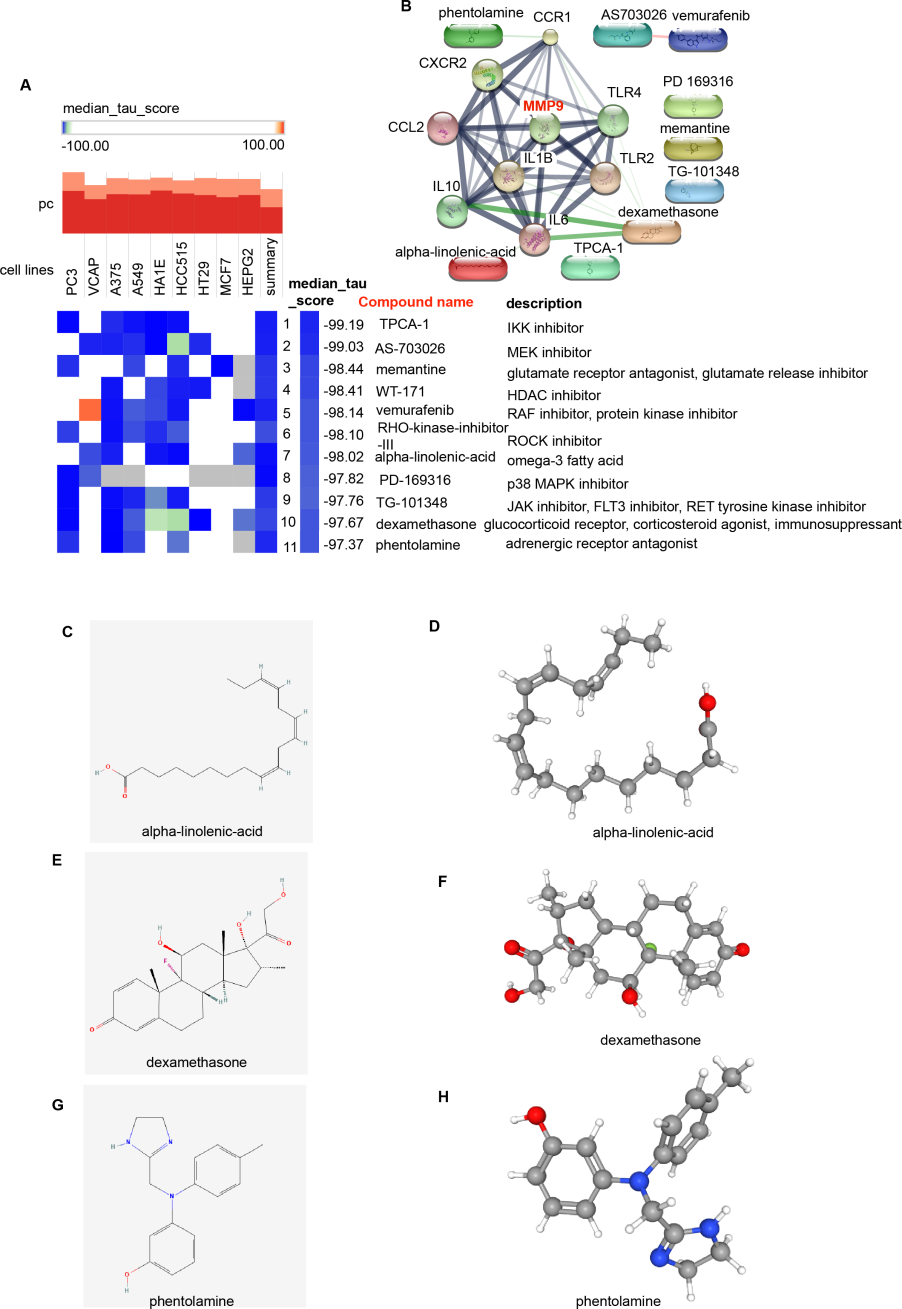
Screening of potential small-molecule compounds for the treatment of *H. pylori* and IBD via cMAP analysis. **(A)** Heatmap showing the top 11 compounds with significantly negative enrichment scores (score < -97) in 10 cell lines and a description of those top 11 compounds via cMAP analysis. We choose Perturbagen Type: compound. **(B)** PPI network diagram of the 11 compounds and Hub genes. **(C, E, G)** Chemical structures of the 3 compounds (alpha-linolenic acid, dexamethasone and phentolamine). **(D, F, H)** 3D structures of the 3 compounds. cMAP, Connectivity Map.

To identify potential small-molecule drugs that target *H. pylori* and IBD, we utilized the CMap method to analyze the Hub genes. A total of 11 potentially effective drugs were predicted (score < −97) (**Figure 7A**; **Supplementary Table 6**), including TPCA-1, AS-703026, memantine, WT-171, vemurafenib, RHO-kinase-inhibitor-III, alpha-linolenic acid, PD-169316, TG-101348, dexamethasone and phentolamine. We further analyzed the interactions between these 11 predicted drugs and Hub genes via the Search Tool for Interactions of Chemicals (STITCH) database (25). The results revealed that alpha-linolenic acid could interact with IL6; dexamethasone could interact with IL10, IL6, IL1B, CCR1, CCL2 and TLR4; and phentolamine could interact with CCR1 (**Figure 7B**). Additionally, the unique chemical and 3D structures of these three drugs (alpha-linolenic acid, dexamethasone and phentolamine) were predicted via the PubChem (**Figures 7C–H**).

### 3.7 Expression of transcription factors in *H. pylori* and IBD

**Figure 8:**
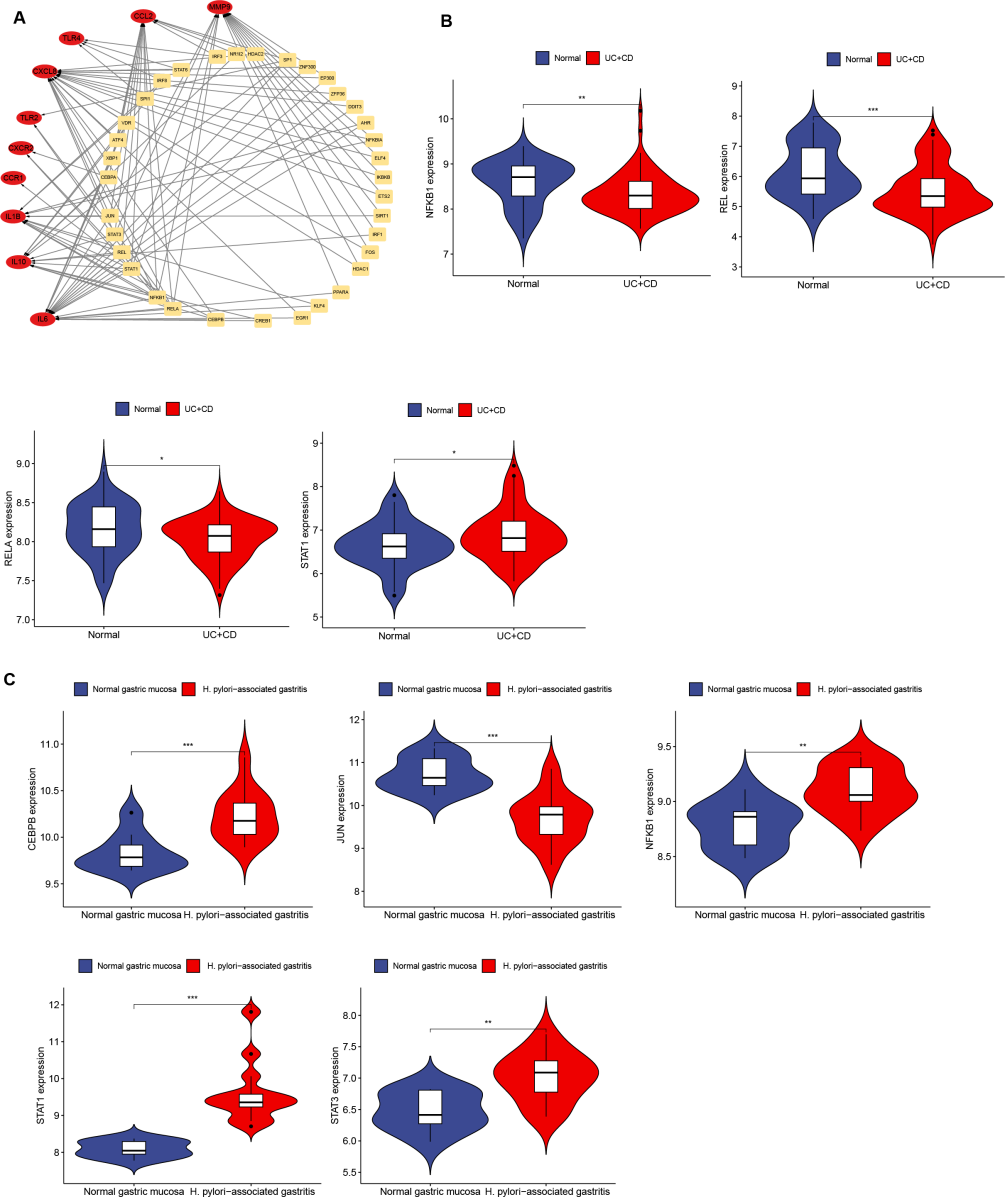
Network interaction between Hub genes and transcription factors and validation in *H. pylori* and IBD. **(A)** Network interaction diagram of transcription factors and Hub genes. **(B, C)** Expression of transcription factors in *H*. *pylori-*infected and IBD patients, with transcription factors showing significant differences. Blue represents the normal group, and red represents the disease group. *p < 0.05; **p < 0.01; ***p < 0.001.

The expression of transcription factors in *H. pylori* and IBD patients was analyzed via TRUST to investigate the interactions of transcription factors with Hub genes. A transcription factor regulatory network was constructed to identify transcription factors that interact with Hub genes (**Figure 8A**; **Supplementary Table 7**). The top 9 transcription factors (those interacting with more than three Hub genes) were selected, including NFKB1, RELA, STAT1, STAT3, JUN, SP1, REL, CEBPA and CEBPB (**Supplementary Table 8**). Further validation of transcription factor expression was conducted in the GSE3365 and GSE233973 datasets. In the GSE3365 dataset, NFKB1, REL and RELA were significantly downregulated, and STAT1 was significantly upregulated (**Figure 8B**); in the GSE233973 dataset, CEBPB, NFKB1, STAT1 and STAT3 were significantly upregulated, and JUN was significantly downregulated (**Figure 8C**).

## 4 Discussion

Studies have shown that IBD is an autoimmune disease and that *H. pylori* infection and colonization have a protective effect against IBD. The mechanisms involved include alterations in inflammatory responses and immune reactions, with improvements in living standards, decreases in *H. pylori* infection rates, and increases in *H. pylori* eradication rates, and the incidence of IBD has gradually increased in some regions (26, 27). Additionally, some animal experiments have confirmed a negative correlation between *H. pylori* infection and the onset of IBD (28, 29). Studies have shown that *H. pylori* activate Toll-like receptors (TLRs), triggering proinflammatory or anti-inflammatory effects, during the progression of *H. pylori* infection. Inflammatory factors (IL-6, IL-8, IL-18, IL-32, IL-1β, TNF-α, CCL2, CCL20, CXCL1 and CXCL10) and immune cells (dendritic cells, regulatory T cells, B cells and M2 macrophages) play key roles in the persistence, differentiation, malignant progression, immune infiltration, immune response and immune evasion associated with *H. pylori*-related diseases, involving various pathogenic mechanisms (30–36).

The pathogenesis of IBD is related to immune system impairment, where activation of the immune system promotes inflammatory responses, leading to tissue damage. Inflammatory factors play important regulatory roles in intestinal mucosal inflammation. Changes in the expression of IL-6, IL-12, IL-23, IL-32, TNF-α and IL-1β promote mucosal inflammation. Therapeutic antibodies and targeted drugs for these factors have been developed and are gradually being used in clinical IBD treatment (37–40). Furthermore, inflammation of the intestinal mucosa is exacerbated by the actions of immune cells, including neutrophils, T lymphocytes, B lymphocytes, macrophages, and dendritic cells (41–45). Therefore, inflammatory factors, immune cells and immune-related genes are key hubs linking *H. pylori* and IBD. This study identified the DEGs commonly expressed in both *H. pylori-*infected patients and IBD patients and explored potential targets to provide a theoretical basis for the management and treatment of patients with both *H. pylori* and IBD.

In this study, we utilized the *H. pylori* and IBD datasets to identify 151 overlapping DEGs. GO and KEGG enrichment analyses indicated that these DEGs were enriched primarily in response to chemokine signaling, the inflammatory response and the immune response. These findings suggest that these DEGs are involved in the regulation of immune and inflammatory responses. Therefore, disruption of the immune system and the inflammatory microenvironment may be major factors in the pathogenesis of *H. pylori* infection and IBD. Using the MCC and degree algorithms, 10 Hub genes were subsequently identified: TLR4, IL10, CXCL8, IL1B, TLR2, CXCR2, CCL2, IL6, CCR1 and MMP-9. GO and KEGG enrichment analyses were performed on these Hub genes, which focused mainly on responses to lipopolysaccharides, responses to molecules of bacterial origin, responses to biotic stimuli and leukocyte migration. These genes were identified as diagnostic markers for *H. pylori* and IBD. Validation using the *H. pylori* and IBD datasets revealed that the expression of Hub genes was upregulated in patients with *H. pylori* infection and IBD, with the expression of MMP-9 significantly upregulated.

Matrix metalloproteinases (MMPs) are a class of glycosylated endopeptidases that degrade extracellular matrix (ECM) molecules, including gelatinase A (MMP-2) and gelatinase B (MMP-9). These MMPs are involved in various inflammatory diseases, such as *H. pylori*-associated gastritis and gastric and duodenal ulcers. Initially, *H. pylori* was shown to activate MMP-9 expression in gastric epithelial cells, contributing to the development of *H. pylori*-related gastric diseases (46, 47). In clinical samples, MMP-9 mRNA expression was found to be significantly upregulated in *H. pylori*-positive patients, with a notable association with CagA status and peptic ulcer disease (PUD) (48). MMP-9 plays a critical role in gastric remodeling in children with *H. pylori*-related gastritis (49). Moreover, studies on single nucleotide polymorphisms (SNPs) of the MMP-9 gene revealed that the rs3918249-rs17576 CG haplotype increased the risk of *H. pylori*-positive PUD (50); SNPs in the MMP-9 gene were also associated with *H. pylori*-positive gastric ulcers (GU) (51). Mechanistically, *H. pylori* lipopolysaccharide (LPS)-induced gastric mucosal MMP-9 release is associated with the activation of the MAPK, ERK, and p38 pathways (52). *H. pylori* can promote the progression of *H. pylori*-related GC by upregulating semaphorin 5A (Sema5A) and regulating the ERK/MMP-9 signaling pathway (53). These studies are consistent with our findings, which demonstrate that MMP-9 is significantly upregulated in *H. pylori* infection in both the training and validation sets. These studies indicate that MMP-9 plays a crucial role in *H. pylori*-related gastric diseases, although the specific molecular mechanisms involved require further investigation.

In the intestinal mucosa of IBD patients, mice, and dogs, MMP-9 has been shown to promote the development of colitis (54–56). In children with UC, its expression increases with the severity of the disease (57). Fibroblasts in IBD continuously express IL-21, which enhances MMP-9 production (58). Some studies have suggested that in mouse models of acute and chronic dextran sulfate sodium (DSS)-induced colitis and acute 2,4,6-trinitrobenzene sulfonic acid-induced colitis, the parameters associated with chronic colitis in MMP-9 knockout mice are similar. Pharmacological inhibition of MMP-9 with bioactive peptides does not improve DSS-induced colitis, suggesting that the upregulation of MMP-9 may be a consequence of intestinal inflammation rather than a cause (59). Mechanistic studies have shown that MMP-9 increases intestinal tight junction (TJ) permeability via the upregulation of myosin light-chain kinase (MLCK) through the p38 kinase signaling pathway in both *in vivo* and *in vitro* models (60). Additionally, MMP-9 regulates intestinal epithelial tight junction barrier function through NF-κB pathway activation of MLCK (61). The long noncoding RNA (lncRNA) growth arrest-specific transcript 5 (GAS5) can regulate MMP-9 expression, mediating tissue damage in IBD patients. Inflammatory tissues show downregulation of GAS5 and upregulation of MMP-9 (62). Furthermore, a mutated CXCL8 protein (G31P) binds to CXCR1/2, rendering CXCL8 inactive, reducing MMP-9 expression, and alleviating colonic fibrosis (63). The upregulation of MMP-9 in IBD is consistent with our validation results, but MMP-9 affects the progression of IBD through multiple pathways. On the basis of the enrichment analysis results in this study, the current research is insufficient to fully explain the pathogenesis of IBD, and further studies are needed to provide a theoretical basis for clinical IBD treatment.

Therefore, MMP-9 may play a significant role in the pathogenesis and progression of both *H. pylori* infection and IBD, and modulating this enzyme could have important implications. The findings of this study provide key clues for further research into the connection between these two diseases and their potential therapeutic targets.

Moreover, MMP-9 is critically involved in a variety of diseases, particularly in cancer. In studies on lymphoma, EGR-1, an early growth response protein 1, is expressed by stromal cells within lymphoma tissues and inhibits the transcriptional activation of the MMP-9 gene in these stromal cells (64). MMP-9 inhibitors have been shown to enhance the functionality of TCR-T cells, which are suppressed by EBV-induced M2 macrophages. The combination of MMP-9 inhibitors with TCR-T-cell therapy has demonstrated effective treatment outcomes for EBV-positive solid tumours (65). Tumor necrosis factor alpha-induced protein 8 (TIPE) has been identified as a promoter of CRC metastasis through its regulatory effects on MMP-9 expression (66). Targeting mTORC1 can facilitate the progression from ductal carcinoma *in situ* (DCIS) to invasive ductal carcinoma (IDC) via the inhibition of MMP-9 (67). In oral squamous cell carcinoma (OSCC), MED1, a subunit of the Mediator complex, activates the transcription of MMP-9 while simultaneously inhibiting CD8+ T-cell antitumor immune responses (68).

Within the context of inflammation, injury repair mechanisms also highlight the significant role played by MMP-9. Recent studies have indicated that unbound bilirubin (UCB) and its derivative dimethyl ester bilirubin (BD1) effectively inhibit the neutrophil-mediated activity of MMP-9 while reducing proinflammatory cytokine gene expression and modulating MAPK pathway signaling to alleviate psoriasis-like skin inflammation in mice (69). Furthermore, the commensal bacterium *Enterococcus faecalis* activates the expression of MMP-9 in host intestinal tissue; pharmacological inhibition of this activation can prevent anastomotic leakage in rat models (70). In cases of ischemic stroke, MMP-9 inhibitors have been shown to specifically reduce the levels of MMP-9, inflammation, and activation while improving neurological function (71). Research on tissue damage and regeneration indicates that liver-specific inhibition of MMP-9 accelerates liver regeneration by 40% following partial hepatectomy; it also prevents proteolytic cleavage of vascular endothelial growth factor-mesenchymal stem cell-derived factor in hepatic tissue, thereby mitigating damage and enhancing regenerative capacity (72). In hydrogel studies, cinnamaldehyde (CA), a specific TRPA1 agonist, is incorporated into a dual-layer hybrid hydrogel (CA@HA-Gel) designed to respond to matrix MMP-9, which inhibits ferroptosis in wound endothelial cells and significantly promotes healing in diabetic wounds (73). In conclusion, MMP-9 plays a critical role in various biological processes, including inflammation, the injury response, tissue regeneration, tumor progression and immune regulation.

In the pathogenesis of *H. pylori* infection and IBD, immune cell infiltration plays an important role. Immune infiltration analysis revealed the relationships between immune cells and cellular molecules in *H. pylori* infection and IBD, which may affect the immune response and inflammatory processes in these diseases. We utilized CIBERSORT to analyze the immune cell infiltration patterns of DEGs in both diseases, and the immune infiltration results were similar. In IBD, immune cell infiltration is increased in CD8+ T cells, monocytes, M0 macrophages, activated mast cells, and neutrophils, whereas memory B cells, resting NK cells, and resting mast cells are decreased. Studies have shown that specific infiltration is dominated by intraganglionic B cells and monocytes in CD, whereas the myenteric plexus (MP) in UC is infiltrated by CD8+ T cells, leading to neuronal apoptosis. In a DSS-induced colitis model, monocyte, B cell, and CD8+ T-cell infiltration also occurs (74). Different clinical outcomes of CD are related to the exhaustion of CD8+ T cells. In active CD, activated CD39+ and CD39+PD-1+ CD8+ T-cell subsets are enriched at low frequencies, whereas during remission, exhaustion-related transcriptional characteristics are upregulated in these subsets (75). Monocytes and macrophages play important roles in regulating the immune response of the intestinal mucosa in IBD. Macrophages produce proinflammatory and anti-inflammatory cytokines, chemokines, toxic mediators, and macrophage extracellular traps (METs), regulating processes such as epithelial cell proliferation, angiogenesis, fibrosis, and the immune response. Additionally, several IBD susceptibility genes related to the maturation and function of macrophages, such as RUNX3, IL21R, GTF2I, and LILRB3, play key roles in the pathogenesis of IBD (76). Synbindin, a classical membrane tethering factor, regulates TLR4 signaling and suppresses the proinflammatory activation of macrophages in response to the microbiota during colitis (77). Moreover, IL-10 is a key regulator of monocyte IL-23 production, providing the basis for targeted therapies such as IL-23p19 and/or IL-1α/IL-1β in IBD patient subgroups (78–80). Our findings, which are consistent with those of other studies, show that the intestinal mucosa in IBD patients contains many activated memory CD4 T cells, M1 macrophages, M2 macrophages, activated mast cells, and neutrophils (81–84). Thus, immune cells play an essential role in regulating immune responses in the intestinal mucosa and provide strategies for the treatment of IBD.

In exploring the relationship between core genes and immune cell infiltration, we emphasize a comprehensive discussion and literature review concerning the association between MMP-9 and the aforementioned significant immune cells. In OSCC, MED1 activates MMP-9 transcription and inhibits the antitumor immune response of CD8+ T cells (68). MMP-9 suppresses the infiltration and cytotoxic activity of CD8+ T cells, thereby providing support for the existence of an inhibitory tumor immune microenvironment (TIME) and resistance to PD-1 inhibitors associated with gain-of-function mutations in CTNNB1 (85). In chronic hepatitis B (CHB), MMP-9 plays an important role in regulating the intrahepatic anti-HBV CD8 T-cell response by mediating the release of soluble CD100 (86). Patients with prostate cancer can increase the expression of MMP-9 in their peripheral blood NK cells and secrete factors that recruit and polarize monocytes (87). Following surgery for acute aortic dissection, platelets release MMP-9 to reprogram monocytes, thereby contributing to postoperative recovery (88). MMP-9 is a central protein related to the immune system and is associated with immune cell infiltration in patients with spinal tuberculosis (STB) (89). In glioblastoma patients, the release of MMP-9 by tumor-infiltrating neutrophils contributes to the efficacy of bevacizumab (90). Therefore, MMP-9 is associated not only with CD8+ T cells, monocytes, and neutrophils but also with other immune cells.

In the analysis of immune infiltration related to *H. pylori* infection, the small sample size of *H. pylori* infection data from the GEO datasets resulted in fewer immune infiltrates than did the results of CIBERSORT. In the future, we plan to conduct transcriptomic sequencing on a large number of *H. pylori*-infected gastric mucosa samples to address the limitations of small sample sizes and perform effective immune infiltration analysis. According to studies on *H. pylori* and immune cell infiltration, *H. pylori* infection induces the infiltration of T lymphocytes and enhances apoptosis through inflammatory responses (91–95). *H. pylori* can induce the expression of cystathionine γ-lyase (CTH) in macrophages, promoting persistent inflammation (96). *H. pylori* infection impacts the TIME, leading to a poorer prognosis in *de novo* gastric diffuse large B-cell lymphoma (gDLBCL) (97, 98). Immune cells play crucial roles in the pathogenesis of *H. pylori*-related diseases.

In addition, this study identified potential small-molecule compounds from the CMap database by screening DEGs in *H. pylori* and IBD and revealed that alpha-linolenic acid, dexamethasone, and phentolamine interact with several Hub genes. We further used the PubChem tool to predict the unique chemical and 3D structures of these three drugs. These small-molecule drugs may improve the pathological conditions of *H. pylori infection* and IBD, providing potential directions for the treatment of these diseases and their associated complications.

The Hub genes were closely related to small molecule drugs and transcription factors. As shown in **Figure 7B**, dexamethasone was closely associated with the Hub genes; however, there was no direct correlation between MMP-9 and dexamethasone, as depicted in **Figure 7B**, although indirect relationships were present. Dexamethasone has been shown to downregulate both MMP-9 expression and oxidative stress levels in mice suffering from eosinophilic meningitis induced by Angiostrongylus cantonensis infection (99). MMP-9 is correlated with the selected small-molecule drugs. Dexamethasone induces the secretion of MMP-9 both *in vitro* and *in vivo*, thereby enhancing the production of mature brain-derived neurotrophic factor (mBDNF), which is essential for synaptic plasticity in adults (100). As shown in **Figure 8**, several transcription factors identified through the selection of Hub genes revealed that in IBD, several transcription factors were significantly downregulated (NFKB1, REL and RELA), whereas STAT1 expression was upregulated. RELA may play an important role in coronary artery disease (CAD) by targeting MMP-9 (101). Additionally, MMP-9 may participate in the development of various diseases through the STAT1-related signaling pathway (89, 102–104). Dexamethasone plays a pivotal role in the clinical management of IBD. Through our bioinformatics exploration, we identified common Hub genes associated with *H. pylori* infection and IBD, particularly MMP-9, which was significantly upregulated under both conditions. On the basis of our literature review informed by bioinformatics, we hypothesize that the activation of this pathway increases the expression of transcription factors, activates MMP-9, and promotes inflammatory responses as well as immune cell activation. Furthermore, the small-molecule drugs we identified may modulate the expression of MMP-9 through various pathways, indicating a need for further investigation.

MMP-9 is a common diagnostic marker for both *H. pylori* and IBD, and although some relevant studies exist in these two diseases, the underlying mechanisms remain unclear. Due to funding limitations, further validation and in-depth exploration of these mechanisms were not conducted. In the future, our research group will further investigate the pathogenesis of these two diseases to provide a theoretical basis and direction for disease prevention and treatment.

## 5 Conclusion

In this study, we revealed that the inflammatory response and immune response may be the common pathogenic mechanisms of *H. pylori* infection and IBD. Additionally, we identified 10 Hub genes (TLR4, IL10, CXCL8, IL1B, TLR2, CXCR2, CCL2, IL6, CCR1 and MMP-9) with significant diagnostic relevance, among which MMP-9 was significantly upregulated in both the training and validation sets for these two diseases. MMP-9 may modulate target genes associated with inflammation and immune responses via transcription factor activation, and the small-molecule drugs identified could serve as a significant strategy for inhibiting MMP-9, thereby providing a scientific rationale for clinical interventions in *H. pylori* and IBD.

## Data Availability

The original contributions presented in the study are included in the article/**Supplementary Material**. Further inquiries can be directed to the corresponding author/s.
